# A deep learning approach to automatic teeth detection and numbering based on object detection in dental periapical films

**DOI:** 10.1038/s41598-019-40414-y

**Published:** 2019-03-07

**Authors:** Hu Chen, Kailai Zhang, Peijun Lyu, Hong Li, Ludan Zhang, Ji Wu, Chin-Hui Lee

**Affiliations:** 1 Center of Digital Dentistry, Peking University School and Hospital of Stomatology & National Engineering Laboratory for Digital and Material Technology of Stomatology & Research Center of Engineering and Technology for Digital Dentistry of Ministry of Health & Beijing Key Laboratory of Digital Stomatology, Beijing, China; 2 Center of Signal and Information Processing (CSIP), School of Electrical and Computer Engineering, Georgia Institute of Technology, Atlanta, GA USA; 3 Department of Electronic Engineering, Tsinghua University, Beijing, China; 4 First Clinical Division, Peking University School and Hospital of Stomatology, Beijing, China

## Abstract

We propose using faster regions with convolutional neural network features (faster R-CNN) in the TensorFlow tool package to detect and number teeth in dental periapical films. To improve detection precisions, we propose three post-processing techniques to supplement the baseline faster R-CNN according to certain prior domain knowledge. First, a filtering algorithm is constructed to delete overlapping boxes detected by faster R-CNN associated with the same tooth. Next, a neural network model is implemented to detect missing teeth. Finally, a rule-base module based on a teeth numbering system is proposed to match labels of detected teeth boxes to modify detected results that violate certain intuitive rules. The intersection-over-union (IOU) value between detected and ground truth boxes are calculated to obtain precisions and recalls on a test dataset. Results demonstrate that both precisions and recalls exceed 90% and the mean value of the IOU between detected boxes and ground truths also reaches 91%. Moreover, three dentists are also invited to manually annotate the test dataset (independently), which are then compared to labels obtained by our proposed algorithms. The results indicate that machines already perform close to the level of a junior dentist.

## Introduction

Human teeth are generally hard substances and do not damage easily; their shapes can remain unchanged after a person’s death without being eroded. Therefore, they play an important role in forensic identification^1–6^. X-ray films obtained from a cadaver’s teeth are usually compared with their dental film records so that even the identity of a deceased person can still be effectively determined. Humans usually have 32 teeth. If all the teeth are screened during comparison, the system will encounter a large computational burden and reduction in accuracy. Segmenting teeth from the X-ray film and performing numbering for each tooth, the testing teeth can be compared only with those having the same numbers in the database, thus the computational efficiency and accuracy can be improved. Further, the oral medical resources are sparse in several developing countries^7^. Dentists usually need to serve numerous patients every day. As an important auxiliary diagnostic tool, a large number of dental X-ray films are photographed daily^8^. Because the film reading work is primarily conducted by dentists, it occupies several valuable clinical hours and may cause misdiagnosis or underdiagnosis owing to personal factors, such as fatigue, emotions, and low experience levels. The work burden of a dentist and the occurrences of misdiagnosis may be reduced if intelligent dental X-ray film interpretation tools are developed to improve the quality of dental care. From this perspective, automatic teeth identification using digitized films is an important task for smart healthcare.

To achieve high-accuracy segmentation and classification in dental films, several scholars have developed image-processing algorithms^9–16^. In their studies, mathematical morphology^10^, active contour^11^ or level-set method^15^ was used for teeth segmentation, while Fourier descriptors^9^, contours^13^, textures^15^ or multiple criteria^16^ were extracted as features, and finally, Bayesian techniques^9^, linear models^12^, or binary support vector machines^13,14^ were used to perform the classification. However, the majority of these algorithms often conduct an image enhancement process before segmentation and feature extraction, and the image features are usually extracted manually. This constitutes a large workload, and the performance of image recognition significantly depends on the quality of the extracted features. Although certain researches achieved satisfactory results, only a few numbers of high-quality images were tested.

Deep learning has developed in recent years, and is capable of automatically extracting image features using the original pixel information as input. These new algorithms significantly reduce the workload of human experts, and can extract certain features that are difficult for humans to recognize. In 2012, a deep convolutional neural network (CNN) achieved satisfactory results in the ImageNet classification work^17^. Afterwards, Regions with Convolutional Neural Network features (R-CNN)^18^, fast R-CNN with spatial pyramid pooling^19,20^, and faster R-CNN with region proposal network^21^ were proposed and obtained increasingly superior results with regard to object detection tasks. Moreover, Inception modules^22^ were also constructed to reduce the computational cost, and Resnet^23^ was proposed to allow training of exceedingly deep networks with more than 100 hidden layers. At present, the deep learning methods based on CNN have become an important methodology in the field of medical image analysis^24,25^. Further, it is expected to aid in teeth detection and numbering tasks in dental X-ray films.

In our previous work^26^, one teeth detection and numbering network based on fast R-CNN was established and it yielded certain preliminary results. To improve the performances, in this study, we propose a deep learning approach for automatic teeth detection and numbering based on faster R-CNN with improved efficiencies and reduced workloads. Prior domain knowledge is also utilized to improve algorithm performance of the baseline faster R-CNN model, which is only a generic tool for general image recognition tasks but does not consider known tooth configuration information.

## Materials and Methods

This study was approved by the bioethics committee of Peking University School and Hospital of Stomatology (PKUSSIRB-201837103). The methods were conducted in accordance with the approved guidelines. The X-ray films used in this study were selected from a database without extraction of patients’ private information, such as name, gender, age, address, phone numbers, etc. All these films were obtained for ordinary diagnosis and treatment purposes. The requirement to obtain informed consent from patients was waived by the ethics committee.

**Figure 1:**
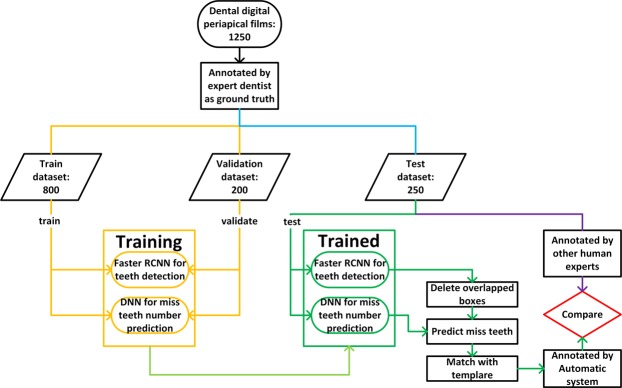
Research work flow.

The overall work flow of this research is illustrated in Fig. 1. A total of 1250 dental X-ray films were collected and separated to form train dataset, validation dataset, and test dataset. Train dataset and validation datasets were used to train a faster R-CNN and a deep neural network (DNN). When testing, images in the test dataset were analyzed via trained faster R-CNN where teeth were detected, and missing teeth were also predicted by the trained DNN. After the post-processing procedure, images were finally annotated automatically, and then compared with the annotations by three dentists.

### Image data and ground truth annotations

**Figure 2:**
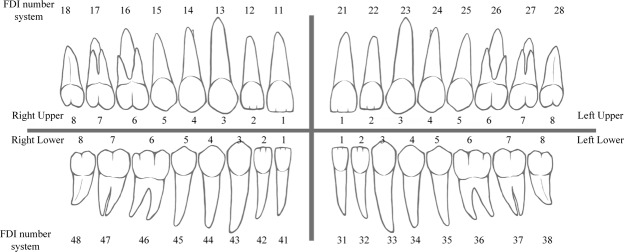
FDI teeth numbering system: 11–18 = right upper 1–8, 21–28 = left upper 1–8, 31–38 = left lower 1–8, 41–48 = right lower 1–8; 1. Central incisor, 2. Lateral incisor, 3. Canine, 4. First premolar, 5. Second premolar, 6. First molar, 7. Second molar, 8. Third molar.

A total of 1,250 digitized dental periapical films were collected from Peking University School and Hospital of Stomatology. Each film was digitized with a resolution of 12.5 pixel per mm at size of approximately (300 to 500) × (300 to 400) pixels and saved as a “JPG” format image file with a specific identification code. These image files were collected anonymously to ensure that no private information (such as patient name, gender, and age) was revealed. Subsequently, an expert dentist with more than five years of clinical experience drew a rectangular bounding box to frame each intact tooth (including crown and root) and provided a corresponding tooth number as ground truth (GT). The Federation Dentaire Internationale (FDI) teeth numbering system (ISO-3950) was used, labeling the upper right 8 teeth as 11–18, upper left 8 teeth as 21–28, lower left 8 teeth as 31–38, and lower right 8 teeth as 41–48 (as shown in Fig. 2). When annotating, the doctor was asked to draw a minimal-size bounding box for each tooth in an image. The coordinates of the points in the image were set as pixel distances from the image’s left top corner, where the tooth bounding box could be recorded via its top left and bottom right corner points (xmin, ymin, and xmax, ymax). A tooth that was truncated at the edge of the image would not be annotated if the truncated portion exceeds 1/2 of the tooth size.

The 1,250 annotated images were randomly divided into 3 datasets: a training set with 800 images, a validation set with 200 images, and a test set with 250 images.

### Neural network model construction, training, and validation

An object detection tool package^27^ based on TensorFlow, with source code, was downloaded from github^28^. Faster R-CNN with Inception Resnet version 2 (Atrous version), which was one of the state-of-the-art object detectors for multiple categories, was selected as the neural network model.

The training process was executed on a GPU (Quadro M4000, NVIDIA, USA), with 8GB memory and 1664 CUDA cores. The algorithms were running backend on TensorFlow version 1.4.0 and operating system was Ubuntu 16.04.

A set of 800 annotated X-ray images was used to train the object recognition faster R-CNN. The input images were resized while maintaining their original aspect ratio, with minor dimension to be 300 pixels. A total of 32 teeth classes were required to be recognized in the X-ray images.

Mean average precision (mAP)^29^ was selected as a metric to measure the accuracy of the object detector during validation process, so as to adjust the train parameters. First, the detected boxes were compared with ground truth boxes, and Intersection-Over-Union (IOU) is defined as:

1 $$IOU=\frac{Are{a}_{DB}\cap Are{a}_{GTB}}{Are{a}_{DB}\cup Are{a}_{GTB}}$$

where $Are{a}_{DB}$ and $Are{a}_{GTB}$ represent the areas of the detected box and its corresponding ground truth box. With the threshold of IOU set to be 0.5, *Precision* and *Recall* are calculated:

2 $$Precision=\frac{TP}{TP+FP}$$

3 $$Recall=\frac{TP}{TP+FN}$$

Where *TP* (True Positive) is the number of objects detected with *IOU* > 0.5, *FP* (False Positive) is the number of detected boxes with *IOU* < = 0.5 or detected more than once, *FN* (False Negative) is the number of objects that are not detected or detected with *IOU* < = 0.5.

For each object class, an Average Precision (*AP*) is defined^29^:

4 $$AP=\frac{1}{11}\sum _{r\in \{0.0,0.1,\ldots ,1.0\}}{p}_{interp}(r)$$

Where ${p}_{interp}(r)$ is the maximum precision for any recall values exceeding *r*^29^:

5 $${p}_{interp}(r)={}_{\tilde{r:}\ge r}{}^{max\,}\,p(\tilde{r})$$

Finally, the mean average precision (*mAP*) is calculated as an average of APs for all object classes:

6 $${mAP}=\frac{1}{{N}_{class}}{\sum }^{}AP$$

After several attempts, the training parameters were adjusted as follows to achieve a high mAP: a batch size of 1, a total of 50000 iterations, an initial learning rate of 0.004 and then reduced to half the rate after 10000 iterations. A pre-trained model on the Coco data set was loaded as a fine tune check point. All other settings were default.

The average training time was approximately 1.1 second per iteration. The total loss dropped from 5.84 to approximately 0.03 after 50000 iterations and mAP on the validation dataset increased to a plateau of approximately 0.80.

### Metrics of performances on test images

After training and validation, the model was tested on the test dataset of 250 images. The detected boxes were evaluated using certain metrics that followed clinical dental considerations.

The boxes detected by the trained faster R-CNN were compared with the ground truth boxes. Each of the *Q* detected boxes was paired with each of the *R* ground truth boxes, and the IOU of each box-pair (detected box - ground truth box) was calculated, forming an IOU matrix of dimension *Q* × *R*.

A box-pair with a value exceeding a threshold of 0.7 in the IOU matrix was considered to be a match. Subsequently, the matched box-pair element was removed from the matrix, and the process was repeated until the max IOU value was under the threshold of 0.7 or no box-pairs existed.

The matched boxes were considered to successfully detect the teeth from the background in the X-ray films. The precision and recall of teeth detection can be calculated as follows:

7 $${Detection}\,{Precision}=\frac{{N}_{match}}{{N}_{DB}}$$

8 $${Detection}\,{Recall}=\frac{{N}_{match}}{{N}_{GTB}\,}$$

Where ${N}_{match}$ is the number of matched box-pairs, ${N}_{DB}$ is number of detected boxes, and ${N}_{GTB}$ is number of ground truth boxes. The *mean IOU* value of the matched boxes, defined below, represents how precise the detected boxes match with the ground truth boxes.

9 $${MeanIOU}=\frac{{\sum }^{}IO{U}_{match}}{{N}_{match}}$$

If a detected box and its matched ground truth box have the same label of a tooth number, it is correctly numbered, meaning a true positive numbering (*TPN*). The precision and recall of teeth numbering can be calculated as follows:

10 $$Numbering\,Precision=\frac{{N}_{TPN}\,}{{N}_{DB}}$$

11 $$Numbering\,Recall=\frac{{N}_{TPN}\,}{{N}_{GTB}}$$

### Postprocessing procedures

To improve the teeth numbering results, certain postprocessing procedures were proposed.

#### Filtering of excessive overlapped boxes

**Figure 3:**
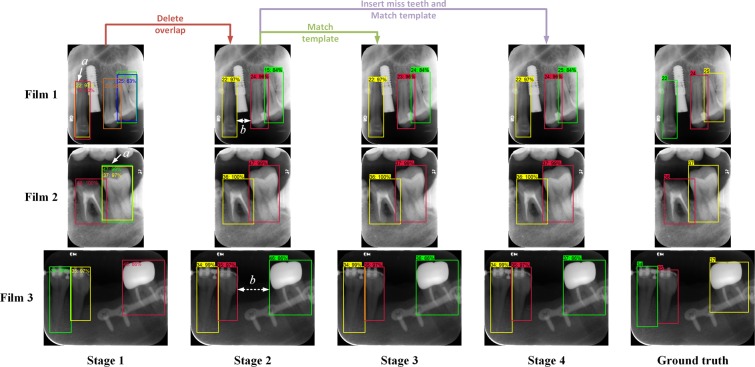
Examples of annotations processing after each stage: (Stage 1) annotated by the trained faster R-CNN, there were certain overlapping boxes (**a**); (Stage 2) after deleting the overlap boxes with lower scores, certain labels of teeth number were incorrect because the neural network confused them with other similar teeth; (Stage 3) the teeth number labels were matched with template for correction, but errors were induced when there were missing teeth (in films 1 and 3), however, the gap between adjacent teeth boxes (stage 2). (**b**) could be treated as a feature to predict the missing teeth; (Stage 4) after inserting the predicted missing teeth and matching with template from (Stage 2), the labels of teeth number were corrected.

The non-maximum suppression algorithm^21^ had been applied for teeth box detection. The overlapped boxes with the same predicted teeth number will be sorted by their probability scores, of which the box with the maximum score will be retained and other boxes that have an IOU (with the maximum score box) larger than the threshold of 0.6 will be deleted. However, the overlapped boxes with a high IOU will not be detected if they are predicted with different numbers (Fig. 3a). To detect these overlapped boxes, IOUs of any pair of boxes in an image were calculated. When an IOU of the box-pair exceeding the threshold of 0.7 is detected, the box with a lower score will be deleted.

#### Application of teeth arrangement rules

After deleting the overlapping boxes, the precision and recall of teeth numbering were still below 0.8, which might be because of the limited number of images trained in this research. However, there are certain rules of teeth arrangement that might help. For an intact dentition with no teeth missing, there are usually 16 teeth in either upper or lower dentition (Fig. 2), with a bilateral symmetry. Using the FDI teeth number system, the arrangement of teeth is numbered as follows: 18–11 for upper right, 21–28 for upper left, 48–41 for lower right, and 31–38 for lower left. Moreover, all these teeth can be classified into six categories: wisdom, molar, premolar, canine, lateral incisor, and central incisor, and teeth in the same category have a high-level of similarity, and also certain degree of similarity can be applied between different categories. These aforementioned prior domain knowledges were taken advantage of to improve the results of teeth detection.

The FDI teeth number system was used as the template, which has an arrangement of “18,17,16,15,14,13,12,11,21,22,23,24,25,26,27,28” for the upper teeth, and “48,47,46,45,44,43,42,41,31,32,33,34,35,36,37,38” for the lower teeth. Because all the teeth in one X-ray image belonged to the same dentition, either upper or lower, the detected box labels should match either the upper or lower teeth template. For example, if the detected box labels in one image was “17,16,14,15,13”, comparing with the upper template, the labels “14,15” would be considered as a wrong arrangement, and it should be corrected to be “17,16,15,14,13”.

When comparing the predicted teeth number list in an image with the template, the predicted list was made to slip in the template from left to right, and a *match score* was calculated at each point:

12 $$Match\,Score=\sum _{X\in \{x|x=T\}}Prediction\,Score\,(X)$$

**Figure 4:**
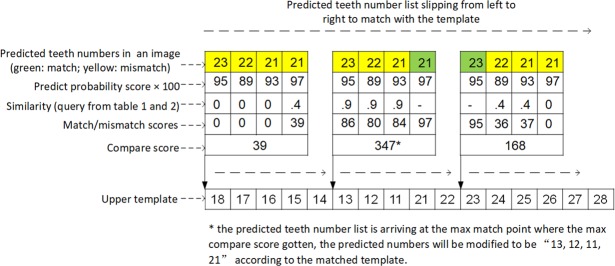
Illustration of the teeth arrangement template-matching algorithm.

Where only the *prediction score* (probability score outputted by the faster R-CNN) of a teeth number (*X*), which matched with the template, i.e., the predicted teeth number of the detected box equals to the teeth number in the template (*x* = *T*), will be summed, as seen in Fig. 4.

If the predicted teeth number does not equal to that in its corresponding template, a weight of mismatch *similarity* between the predicted tooth and template tooth should be applied to calculating a “*mismatch score*.” First, all the teeth were classified into several categories according to their appearances (Table 1). For teeth in the same dentition, the mismatch similarity matrix was set according to an expert dentist’s experience to provide the value of similarity between categories (Table 2). The *mismatch score* was calculated as follows:

13 $$Mismatch\,Score=\sum _{X\in \{x|x\ne T\}}Prediction\,Score\,(X)\ast similiarity\,(X,T)$$

where the *prediction score* was multiplied with a *similarity* value, which can be inferred from Table 2, according to the category of the predicted tooth number (*X*) and its corresponding template tooth number (*T*) in Table 1. Finally, a *comparison score* was defined to be the sum of the match score and mismatch score:

14 $$Comparison\,Score=Match\,Score+Mismatch\,Score$$

**Table 1:** Categories of teeth.

Object		Value															
Upper dentition	Teeth ID	18	17	16	15	14	13	12	11	21	22	23	24	25	26	27	28
	Category	W	M	M	P	P	Ca	La	Ce	Ce	La	Ca	P	P	M	M	W
Lower dentition	Teeth ID	48	47	46	45	44	43	42	41	31	32	33	34	35	36	37	38
	Category	W	M	M	P	P	Ca	I	I	I	I	Ca	P	P	M	M	W

W = Wisdom, M = Molar, P = Premolar, Ca = Canine, La = Lateral Incisor, Ce = Central Incisor, I = Incisor.

**Table 2:** Similarity matrix between teeth categories for mismatches.

Upper dentition							Lower dentition					
	W	M	P	Ca	La	Ce		W	M	P	Ca	I
W	0.9	0.8	0	0	0	0	W	0.9	0.7	0	0	0
M	0.8	0.9	0	0	0	0	M	0.7	0.9	0	0	0
P	0	0	0.9	0.6	0.4	0.4	P	0	0	0.9	0.5	0.3
Ca	0	0	0.6	0.9	0.6	0.8	Ca	0	0	0.5	0.9	0.5
La	0	0	0.4	0.6	0.9	0.8	I	0	0	0.3	0.5	0.9
Ce	0	0	0.4	0.8	0.8	0.9						

W = Wisdom, M = Molar, P = Premolar, Ca = Canine, La = Lateral Incisor, Ce = Central Incisor, I = Incisor.

The slipping label list will arrive at a most matched point where the max *comparison score* was obtained and the template will be used to correct the prediction number list at this point (Fig. 4).

#### Prediction of missing teeth

In cases of missing teeth, the scheme of the FDI system will never be matched, unless there are placeholders for the missed teeth in the predicted teeth number list. As shown in Fig. 3b, there are usually gaps between adjacent detected boxes where missing teeth existed, thus the horizontal distance of adjacent box margins is one of the key features to predict missing teeth. However, the gap of missing teeth may disappear when the adjacent teeth have a high degree of incline, where the distance of the center of the adjacent box should be considered as another key feature to predict the missing teeth.

A simple deep neural network classifier with two fully-connected hidden layers (10 neural units each) was set up. The horizontal margin distance and center distance of two adjacent boxes were used as the input features, while the missing teeth number (ranges from 0 to 3, as observed in the train dataset) was set as the label to predict. After training using the same train set of 800 images with 100 epochs, a precision of 0.981 was achieved on the validation dataset. Subsequently, the place holder “M”s were placed where the missing teeth were predicted, “17,16,M,M,13” for example, before matching with the template. The similarity of placeholder “M” with its corresponding template tooth number was set to 0 when calculating the comparison score.

### Comparison with human experts and our previous fast R-CNN method

To evaluate the performance level of the developed teeth detection system, three expert dentists (A, B, and C) were invited to conduct the annotation work on the test dataset. Experts A had approximately three-year experience of observing dental periapical X-rays, and B had approximately two years of experience, while C had approximately four years of experience. The rules of human annotation were set as follows: (1) drawing a minimum-sized bounding box of each tooth in the images, and (2) using the FDI numbering system. Besides, some ground truth annotations in the images of the train dataset were shown as examples to the dentists, from which they could learn how to do annotations. Any modification was allowed during or after each annotation, and the experimenter reviewed the annotations to observe and correct possible mistakes before final submission. The annotations by the dentists were matched with the ground truth data to calculate the precisions, recalls, and IOUs.

## Results

**Figure 5:**
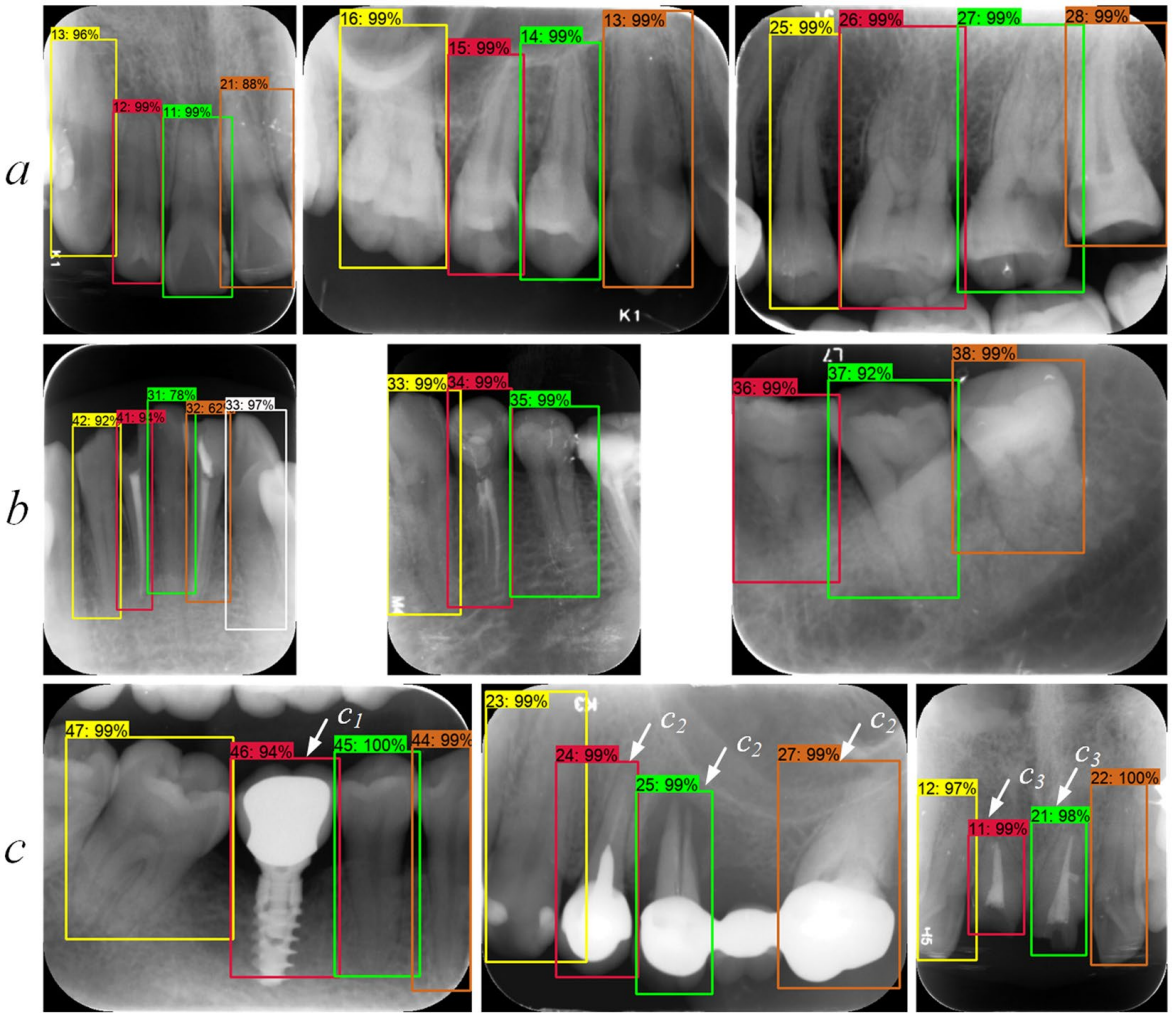
Sample images correctly annotated by neural networks on test dataset: (**a**) upper teeth and (**b**) lower teeth, including incisors, canines, premolars, and molars; (**c**) some complicated cases, including (c_1_) implant restoration, (c_2_) crown and bridge, (c_3_) defected teeth.

The precision, recall, and IOU after each stage are shown in Table 3. Certain examples of true positive teeth detection boxes and teeth numbering labels were shown in Fig. 5, including certain complicated cases such as implant restorations, crowns and bridges, and defected teeth. The results of the expert controls are shown in Table 3, in the column “Human Experts.” Expert C, who was more experienced than experts A and B, achieved higher accuracy. The train and validation datasets were also used to train our previous fast R-CNN network^26^, and the performances regarding test images are also shown in Table 3, in the column “Prior work”, demonstrating a lower accuracy.

**Table 3:** The precision, recall, and IOU of detected box on test dataset.

Object	AS*				Human Experts			Prior work^26^
	stage 1**	stage 2**	stage 3**	stage 4**	A	B	C	
Test images	250	250	250	250	250	250	250	250
GT* boxes exist	871	871	871	871	871	871	871	871
Box detected	953	868	868	868	869	866	873	822
Detection prec*	0.900	0.988	0.988	0.988	0.993	0.991	0.995	0.838
Detection recall	0.985	0.985	0.985	0.985	0.991	0.985	0.998	0.791
Mean IOU	0.91 ± 0.04	0.91 ± 0.04	0.91 ± 0.04	0.91 ± 0.04	0.92 ± 0.05	0.90 ± 0.05	0.92 ± 0.05	0.81 ± 0.06
Numbering prec*	0.715	0.797	0.897	0.917	0.938	0.930	0.975	0.771
Numbering recall	0.782	0.794	0.894	0.914	0.936	0.924	0.977	0.728

*AS = our automatic teeth detection and numbering system, GT = ground truth, prec. = precision.

**Stage 1: teeth bounding boxes detected by trained faster R-CNN; stage 2: after deleting overlapped boxes; stage 3: after matching with template; stage 4: after predicting missing teeth and matching with template.

**Figure 6:**
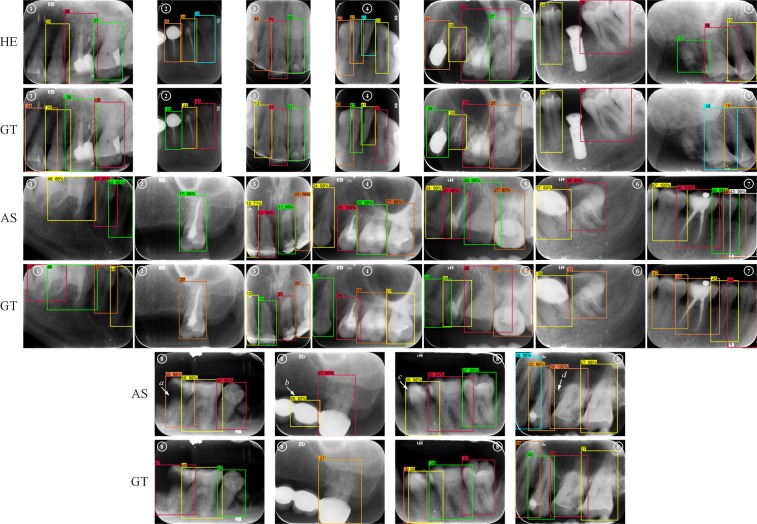
Examples of mismatch annotations on test dataset: (HE) annotations by human expert, (GT) ground truth, (AS) annotations by our automatic system, (1–9) mismatch type 1–9; complicated cases in type 8 such as (**a**) severe decay, (**b**) pontic of long bridge, (**c**) teeth overlap, and (**d**) extracted tooth gap closed by orthodontic therapy.

The mismatch annotations, both produced by our automatic system (AS) and human experts (HE), were analyzed. There were eight types (①–⑧) of mismatches in the AS annotations and seven types (①–⑥, ⑨) in the HE annotations, of which six types were common in both annotations (Table 4, Figs 6). All these mismatches can be explained as: 1 certain bounding boxes, primarily for partially truncated or overlapped teeth, were not detected; 2 primarily for the posterior teeth, the left teeth were numbered to be right teeth, and vice-versa, for e.g. ‘25’ labeled as ‘15’; 3 primarily for the anterior teeth, the teeth with similar shapes were sometimes confused with each other, e.g. ‘12’, ‘11’, ‘21’, and ‘22’ were likely to mixed and wrongly numbered; 4 there were certain missing teeth that were not recognized and so the afterward or forward teeth numbers were wrong; 5 the region of the detected box had a low IOU with ground truth box that was less than the threshold of 0.7; 6 there were also several controversial labels that could not be defined as right or wrong, because the teeth features presented in these images were not sufficient and even the ground truth labels could not be guaranteed; 7 few boxes for teeth with two ‘half tooth’ in them were generated by faster R-CNN, which were misunderstood as one ‘intact tooth’; 8 our system failed to number the teeth correctly in certain complicated cases, such as heavily decayed teeth, large overlaps, big prosthodontic restorations, and orthodontic treatment finished after teeth extraction, where the FDI teeth number template could not be applied; 9 certain heavily defected teeth with only small residual roots were annotated by the dentists correctly while ignored by the ground truth.

**Table 4:** Number of images with mismatch annotations.

Mismatch type	AS*	Expert A	Expert B	Expert C
① Box undetected	5	3	4	1
② Reversed left and right	1	4	1	0
③ Confusion with similar teeth	8	8	10	2
④ Missing teeth not recognized	5	5	3	1
⑤ Poor region match	4	3	6	2
⑥ Unclear labels	5	4	5	4
⑦ Inter-teeth boxes	4	0	0	0
⑧ Failure in complicated cases	5	0	0	0
⑨ Objects detected more than GT*	0	2	2	2
Total	37	29	31	12

^*^AS = our automatic teeth detection and numbering system; GT = ground Truth.

## Discussion

Periapical films can capture images of intact teeth, including front and posterior, as well as their surrounding bone, which is exceedingly helpful for a dentist to visualize the potential caries, periodontal bone loss, and periapical diseases. Bitewing films, which were primarily researched in the previous studies^9–15^, can only visualize the crowns of posterior teeth with simple layouts and considerably less overlaps. From this point of view, it is more difficult to detect and number teeth in periapical films than in bitewing films. Moreover, the mathematical morphology method used in Said’s research^10^ exhibited considerably complicated procedures and thereby less automation efficacy. Jader *et al*.^30^ used Mask R-CNN to conduct the deep instance segmentation in dental panoramic X-ray images, which could outline the profile of each tooth. However, the annotation work for instance segmentation is of high cost, and only less than 200 images were annotated to train their network, resulting in certain rough segmentations. Images used in our research were all obtained from ordinary clinical work, which were randomly selected from the hospital database without screening. As a result, there were several complicated cases, including filled teeth, missing teeth, orthodontic treated teeth with premolars extracted, embedded teeth, retained deciduous teeth, root canal treated teeth, residual roots, implant restored teeth, and teeth with crowns and bridges, which presented challenges. However, with the exceptional performance of deep learning neural network and our post-processing procedures, a satisfactory result was achieved.

As observed in this research, our prior domain knowledge considerably helped in the teeth numbering, with almost 10% increase of the precision and recall. Since there is a high-level similarity of teeth in the same category, e.g., 17, 16, 26, 27 all belong to upper molars, the neural network always confused and misclassified them into each other. The rules of teeth arrangement regarding an image, which are important to number the teeth, were not properly learned by the object detection network. This was not surprising because there was almost no consideration of relationships between the detected boxes in this object detection neural network. The FDI teeth numbering system provided a sequence of teeth number, which was used as a template to correct the wrongly numbered teeth, while a similarity matrix was established to provide certain reasonable tolerance for the mismatched numbers. With these postprocessing of predicted numbers, the precision and recall of teeth numbering evidently improved. However, the similarity matrix was constructed totally based on a dentist’s experience. Although the result was satisfactory, there is still room to improve the performance if more values are tested and better ones are selected for the matrix.

Before matching with the template, the status of missing teeth in the image should be considered. Under conditions of missing teeth, place holders that equal to the number of missing teeth should be inserted into the predicted teeth number sequences at correct points. In this research, a neural network with simple architecture was established to predict the missing teeth number between two adjacent teeth, and only two features were selected as the input. There is also considerable room to improve the missing teeth prediction, for example, the gray value between teeth should be concerned and a flexible algorithm allowing calculation of possibility scores of different predicted missing teeth numbers may help to increase the precision further.

The high precision, recall, and IOU of the detected boxes matching with ground truth boxes demonstrate the neural network system’s ability to distinguish the “shape” of teeth from the background correctly. The region proposal work was so good that the predicted bounding box areas were nearly the same with the ground truth ones. These automatically detected teeth bounding boxes are of significant value to extract teeth out of the dental X-ray images, which means a large number of teeth can be automatically segmented from a big database of hospital dental X-ray films and presented for further analysis.

The performances in this study were considerably better than our previous work based on fast R-CNN. The improvement of the neural network architecture and postprocessing procedures were significant. The precision, recall, and IOU of annotations made by our system were exceedingly close to that made by a junior dentist. The analysis of mismatch annotations demonstrates that most (six) types of mismatches occurred in annotations by both our automatic system and human experts, implying that our system made mistakes similar to humans, especially in less complex cases. However, the automatic system failed in certain subtle cases that can be easily resolved by human experts. On the contrary, human experts tended to be careless in certain simple cases, where left posterior teeth were labeled to be right ones or vice versa. Errors were not realized even after a comprehensive review of the annotations. In real clinical situation, there may not be sufficient time for the dentists to review their reports carefully, where errors would occur. Thus, it will be a significant help if a well-developed neural network system can be used to assist the dental X-ray diagnosis work.

Although the faster R-CNN network achieved satisfactory results in this research, there were also certain failed cases that recognized two ‘half tooth’ as an intact tooth. This is an inherent drawback of Convolutional Neural Network that does not concern the spatial relationship between image features. While the recently proposed capsule network^31^ may provide a solution to this problem. More types of neural networks and architectures should be tested in future research and a better method might be obtained to improve the teeth detection results.

## Conclusions

In this study, faster R-CNN performed exceptionally well regarding teeth detection, which located the position of teeth precisely with a high value of IOU with ground truth boxes, as well as good precision and recall. However, the precision and recall of the classification work that provided each detected tooth an FDI number was unsatisfactory until certain postprocessing procedures were applied. Our prior domain knowledge, especially regarding teeth arrangement rules and similarity matrixes, played an important role in promoting the teeth numbering accuracies, with an increase in more than 10% of the precision and recall. Finally, the performances of our proposed automatic system were very close to the level of a junior dentist who was selected as a control in this study.

## Supplementary information

Ethics File

## Data Availability

The data that support the findings of this study are available from Peking University School and Hospital of Stomatology but restrictions apply to the availability of these data, which were used under license for the current study, and so are not publicly available. Data are however available from the authors upon reasonable request and with permission of Peking University School and Hospital of Stomatology.
