# Supplementary material for: A deep learning approach to automatic teeth detection and numbering based on object detection in dental periapical films
Source: Sci Rep. 2019 Mar 7;9:3840. doi: 10.1038/s41598-019-40414-y (PMC6405755; doi:10.1038/s41598-019-40414-y)
Supplement: Supplementary file 1 — Ethics File [file 41598_2019_40414_MOESM1_ESM.pdf]

北京大学口腔医院生物医学伦理委员会
伦理审查批件

伦理审查批件号：PKUSSIRB-201837103

受理号	2018-02-37-18		
项目名称	口腔 X 线图像智能识别算法研究及应用评价		
项目负责人	陈虎		
审查类别	初始审查	审查方式	快审
审查日期	2018-04-03	审查地点	
审查委员	冯海兰、周永胜、甘业华、赵玉鸣		
审查文件	详见附件“审查文件清单”		

审查意见：

依据《中华人民共和国执业医师法》、《医疗机构管理条例》、《药物临床试验质量管理规范》，《涉及人体的生物医学研究伦理审查办法》（试行）、《药物临床试验伦理审查工作指导原则》、世界医学会《赫尔辛基宣言》、世界卫生组织《生物医学研究审查伦理委员会操作指南》、国际医学科学组织委员会《涉及人的生物医学研究国际伦理准则》等法律、法规、规章、规范性文件和国际准则，经本伦理委员会审查，同意按研究方案开展本项研究。

请遵循伦理委员会批准的方案开展临床研究，保护受试者的健康与权利。

1. 研究过程中若变更主要研究者，对临床研究方案、知情同意书、招募材料等的任何修改，请提交修正案审查申请；
2. 发生严重不良事件，请及时提交严重不良事件报告。紧急报告之后，请尽快提交详细的严重不良事件随访报告；
3. 当出现任何可能显著影响试验进行、或增加受试者危险的情况时，请立即向伦理委员会提交书面报告；
4. 研究者没有遵从方案开展研究，可能对受试者的权益/健康、以及研究的科学性造成不良影响，请提交违背方案报告；
5. 申请人暂停或提前终止临床研究，请及时提交暂停/终止研究报告。

持续审查频率	一年	注：请在截止日期前至少 1 个月提交研究进展报告。
批件有效期	一年	注：本批件从批准之日起开始生效，效期为 1 年，请在有效期满前一个月提交持续审查报告或结题报告，换取新的有效批件。
研究总结报告	研究结束时，请提交总结报告。	
主任委员签字<br>签署日期	冯海兰 2018.4.9	

北京大学口腔医院生物医学伦理委员会办公室：北京大学口腔医院 行政楼 406（中关村南大街 22 号）
电话：010-82195759 Email: keyanchuethics@163.com
